# Characterization of 15 CYP2J2 variants identified in the Chinese Han population on the metabolism of ebastine and terfenadine *in vitro*

**DOI:** 10.3389/fphar.2023.1186824

**Published:** 2023-05-23

**Authors:** Li-Li Zou, Fang-Ling Zhao, Yu-Ying Qi, Shuang-Hu Wang, Quan Zhou, Pei-Wu Geng, Yun-Fang Zhou, Qing Zhang, Hao Chen, Da-Peng Dai, Jian-Ping Cai, Fu-Sui Ji

**Affiliations:** ^1^ The Key Laboratory of Geriatrics, Beijing Hospital, National Center of Gerontology, Institute of Geriatric Medicine, Chinese Academy of Medical Sciences, Beijing, China; ^2^ The Laboratory of Clinical Pharmacy, The Sixth Affiliated Hospital of Wenzhou Medical University, The People’s Hospital of Lishui, Lishui, China; ^3^ Department of Cardiology, Beijing Hospital, National Center of Gerontology, Institute of Geriatric Medicine, Chinese Academy of Medical Sciences, Beijing, China

**Keywords:** CYP2J2, polymorphism, allelic variant, catalytic activity, Chinese Han

## Abstract

Genetic polymorphism of the cytochrome P450 (CYP) gene can significantly influence the metabolism of endogenous and xenobiotic compounds. However, few studies have focused on the polymorphism of *CYP2J2* and its impact on drug catalytic activity, especially in the Chinese Han population. In this study, we sequenced the promoter and exon regions of *CYP2J2* in 1,163 unrelated healthy Chinese Han individuals using the multiplex PCR amplicon sequencing method. Then, the catalytic activities of the detected CYP2J2 variants were evaluated after recombinant expression in *S. cerevisiae* microsomes. As a result, CYP2J2*7, CYP2J2*8, 13 variations in the promoter region and 15 *CYP2J2* nonsynonymous variants were detected, of which V15A, G24R, V68A, L166F and A391T were novel missense variations. Immunoblotting results showed that 11 of 15 CYP2J2 variants exhibited lower protein expression than wild-type CYP2J2.1. *In vitro* functional analysis results revealed that the amino acid changes of 14 variants could significantly influence the drug metabolic activity of CYP2J2 toward ebastine or terfenadine. Specifically, 4 variants with relatively higher allele frequencies, CYP2J2.8, 173_173del, K267fs and R446W, exhibited extremely low protein expression and defective catalytic activities for both substrates. Our results indicated that a high genetic polymorphism of *CYP2J2* could be detected in the Chinese Han population, and most genetic variations in *CYP2J2* could influence the expression and catalytic activity of CYP2J2. Our data significantly enrich the knowledge of genetic polymorphisms in *CYP2J2* and provide new theoretical information for corresponding individualized medication in Chinese and other Asian populations.

## 1 Introduction

Cytochrome P450 (CYP) enzymes, as membrane-bound hemoproteins, play an important role in drug metabolism, cellular metabolism, and homeostasis. Approximately 70%–80% of clinical drug metabolism and elimination can be attributed to one or more of the various CYP superfamilies (Tao et al., 2020; Song et al., 2021; Zhao et al., 2021). CYP2J2, a member of the CYP450 enzyme family, is a heme-containing epoxygenase that is responsible for olefin epoxidation of endogenous polyunsaturated fatty acids (Spiecker et al., 2004; Sisignano et al., 2020; Ding et al., 2021). This CYP enzyme is involved in many pathological processes, such as coronary artery disease, hypertension, cerebrovascular diseases, diabetes and cancer (Jiang et al., 2005; Li et al., 2012; Azevedo et al., 2016; Das et al., 2020). Although CYP2J2 was first discovered in the liver, it has been demonstrated to be an extrahepatic CYP450 that is predominantly expressed in the cardiovascular system (Michaud et al., 2010; Zanger and Schwab, 2013). CYP2J2 is also distributed in kidneys, lungs, brain, small intestines, gastrointestinal tissues, etc., (Michaud et al., 2010; Das et al., 2020).

The *CYP2J2* gene, located on human chromosome 1p31.3–p31.2, spans approximately 40.3 kb and contains nine exons and eight introns (Wu et al., 1996; Murray, 2016). At least nine *CYP2J2* variants have been elaborated in the Pharmacogene Variation (PharmVar) Consortium, including CYP2J2*2 to *10 (T143A, R158C, I192N, D342N, N404Y, −76G>T, G312R, P351L and P115L, respectively) (King et al., 2002; Spiecker et al., 2004; Gaedigk et al., 2018). Previous studies confirmed that distinct racial populations displayed different *CYP2J2* polymorphisms and allele frequencies (Berlin et al., 2011). For example, the CYP2J2*7 (−76G>T) variant occurred with significantly different frequencies among ethnic populations, with allele frequencies of 4%, 8%, and 17% in Koreans, white people, and Africans, respectively (King et al., 2002; Spiecker et al., 2004; Lee et al., 2005; Wang et al., 2006). Interethnic comparison revealed that allele frequencies of wild-type *CYP2J2* (CYP2J2*1) in East Asians and Caucasians were higher than in Africans (Li et al., 2021).

China, one of the largest countries in the world, is a multinational country with more than 1.4 billion people, but few studies have focused on *CYP2J2* polymorphisms in the Chinese population. Previous studies have identified the CYP2J2*7 variant with allelic frequencies of 2.6%, 3.5%, 4.5%, and 12% in 384 Chinese Han, 100 Chinese Uyghur, 100 Chinese Zhuang ethnic people and 200 Chinese Han individuals with premature MI, respectively (Wang et al., 2006; Liu et al., 2007; Zhang et al., 2016a; Zhang et al., 2016b). CYP2J2*7, *8 (G312R), N190S, R200C, Y203C and A355T were found in 100 Chinese Uyghur; V188I was detected in 100 Chinese Wa; and CYP2J2*7 and M116V were identified in 100 Chinese Tibetan (Zhang et al., 2016a; Zhang et al., 2016b; Cao et al., 2018). Recently, some novel *CYP2J2* variants have been listed in the aggregation resources of the Genome Aggregation Database (gnomAD v3.1.1 database) (Karczewski et al., 2020). However, most *CYP2J2* polymorphism studies in the Chinese population have only focused on the typical defective alleles CYP2J2*7 and/or CYP2J2*8 in relatively small sample sizes. There is a lack of large-scale studies for the Chinese Han, the largest ethnic group in China. Therefore, it is necessary to fully investigate *CYP2J2* polymorphisms and discover specific rare variants in the Chinese Han population.

Although CYP2J2 expression in the liver and intestine accounts for only 1%–2% of CYP isoforms, the CYP2J2 enzyme plays an important role in metabolizing endogenous and xenobiotic compounds (Paine et al., 2006; Yamazaki et al., 2006). Specifically, CYP2J2 has been reported to be the primary CYP enzyme involved in the metabolism reactions of approximately 3% of drugs used today, such as the metabolism of albendazole, fenbendazole and terfenadine (Matsumoto et al., 2002; Lee et al., 2012; Ren et al., 2013; Lu et al., 2014; Solanki et al., 2018; Zhao et al., 2021). Furthermore, previous studies confirmed that CYP2J2 showed a unique metabolism profile compared with CYP3A4/5 for ritonavir metabolism and dominated the hydroxylation of rivaroxaban compared with multiple CYPs (Kaspera et al., 2014; Zhao et al., 2022). However, the catalytic activities of unassigned CYP2J2 genetic variants have not been elucidated. Because of its indispensable role in drug metabolism, exploring the catalytic activities of CYP2J2 variants also becomes an important factor for interindividual and interethnic variability in the dosage of drugs metabolized by CYP2J2.

Herein, we systematically analyzed the promoter and exon regions of the *CYP2J2* gene in 1,163 unrelated healthy individuals of Chinese Han ethnicity using a multiplex PCR amplicon sequencing method and identified CYP2J2*7, CYP2J2*8, 13 *CYP2J2* promoter variants and 15 unassigned *CYP2J2* nonsynonymous variants in the Chinese Han population. We confirmed that most of the identified *CYP2J2* allelic variants in exons changed CYP2J2 protein expression and the metabolic activities of CYP2J2 probe drugs (including ebastine and terfenadine) *in vitro*.

## 2 Materials and methods

### 2.1 Chemicals and material

The FinePure Universal DNA Purification Kit was purchased from GENFINE (Beijing, China). The *CYP2J2* Human Tagged ORF Clone (RC207417) was purchased from Origene (United States). *Saccharomyces cerevisiae* strain YPH499 was obtained from ATCC (VA, United States). The yeast expression vector pESC-URA was purchased from Stratagene (United States). The following primary antibodies were used: rabbit polyclonal anti-CYP2J2 antibody (Abcam, United Kingdom), mouse monoclonal anti-OR antibody (Santa Cruz Biotechnology, United States) and corresponding secondary antibodies (Proteintech, China). Ebastine and terfenadine were purchased from Tokyo Chemical Industry. Hydroxyebastine, terfenadine alcohol and midazolam were purchased from Santa Cruz and TRC. The NADPH-regenerating system was purchased from Promega (United States). High-pressure liquid chromatography-grade solvents were purchased from Fisher Scientific Co., (United States). All the other chemicals and solvents were commercially available at analytical grade or the highest grade.

### 2.2 Multiplex PCR amplicon sequencing of CYP2J2

**FIGURE 1:**
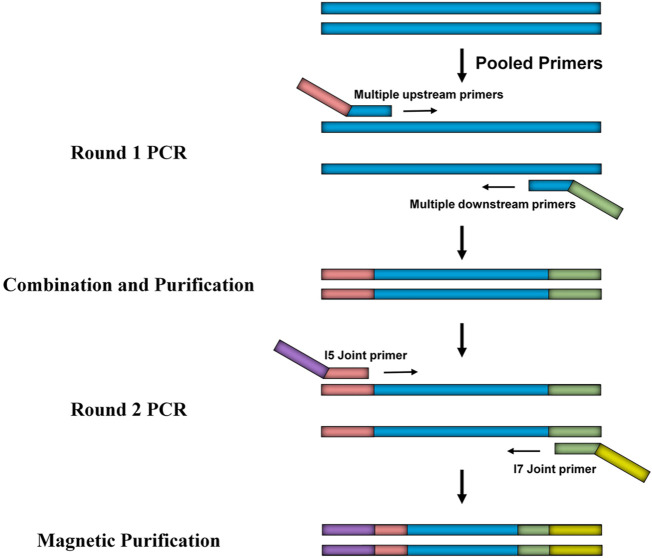
Schematic diagram of the developed multiplex PCR amplicon sequencing method. The method includes two rounds of PCR amplification in which the first round of PCR is for the multiplex amplification of the promoter region and exons of the *CYP2J2* gene, and the second round of PCR is for library construction. Universal primers used in round 1 and round 2 are illustrated as pink, green, purple, and yellow bars.

To systematically investigate CYP2J2 polymorphisms, multiplex PCR amplicon sequencing was developed in this study. As shown in Figure 1, the promoter and 9 exons of the *CYP2J2* gene were amplified in the first round of PCR using pooled primers, which are listed in Supplementary Table S1. After purification with AMPure XP beads (Beckman, United States), pooled amplicons were used for library construction with i5 or i7 containing universal primers in the second round of PCR amplification. The barcoded library was then quantified with a Qubit 3.0 Fluorometer (Thermo Fisher Scientific, United States) and Agilent 2,100 Bioanalyzer system (Agilent, United States) was used to measure the concentration and length of library fragments (from 270 to 420 bp). Qualified libraries were then sequenced on a NovaSeq 6,000 (Illumina, United States) with a paired-end 150 sequencing strategy by iGeneTech Co. Then, FastQC (Version 0.11.9) and Annovar software were applied to obtain high-quality reads and annotate the data based on the reference genome GRCh38 (Wang et al., 2010). Then, the following filtering parameters were included for the selection of genetic variations with high sequencing quality: the sequencing depth was above 50, and the detected frequency of samples was within 0.4–0.6 or 0.9–1.0 for heterozygotes or homozygotes, respectively.

### 2.3 DNA extraction and genotyping

This study was approved by the Ethics Committee of Beijing Hospital institutions. All 1,163 unrelated healthy Chinese Han subjects were recruited during health examinations in the Department of Cardiology of Beijing Hospital and signed written informed consent forms before the blood samples were collected. Genomic deoxyribonucleic acid (DNA) was extracted from blood leukocytes using the FinePure Universal DNA Extraction Kit according to the manufacturer’s manual. Multiplex PCR amplicon sequencing was used to analyze the promoter region and 9 exons of the *CYP2J2* gene in DNA samples. The obtained variation information was then compared with named *CYP2J2* alleles that were assigned by PharmVar Consortium or listed in aggregation resources of the Genome Aggregation Database (Gaedigk et al., 2018; Karczewski et al., 2020) to obtain the genotype of tested individuals. For variations not included in the *CYP2J2* allele list, the promoter region or exons of *CYP2J2* in corresponding subjects were amplified and sent to BioMed Biotech Company for bidirectional Sanger sequencing verification. Detailed primer information for PCR amplification and sequencing is shown in Supplementary Table S2.

### 2.4 Expression of recombinant CYP2J2 variants in *Saccharomyces cerevisiae*

Complete ORFs of CYP2J2*1 and its variants were amplified from the *CYP2J2* Human Tagged ORF Clone by overlap extension PCR amplification using PrimeSTAR MAX high-fidelity DNA polymerase (Takara Bio, Japan) and gene-specific oligonucleotides with the forward primer 2J2-UF (5′-AAG​T*GAA​TTC*ATG​CTC​GCG​GCG​ATG​GGC-3′, introducing an EcoRI site), the reverse primer 2J2-UR (5′-ACT​C*AGA​TCT*TTA​CAC​CTG​AGG​AAC​AGC​GCA-3′, introducing a Bglll site) and the mutation-containing upper or lower primers listed in Table 1 (Dai et al., 2013). Then, the yeast expression vector pESC-OR (obtained by inserting NADPH-CYP450 OR into pESC-URA) and ORF fragments of the *CYP2J2* variants were digested by EcoRI and Bglll and ligated at 16 C for 30 min with Ligation Mix ligase (Takara Bio, Japan). After sequencing verification, the expression plasmids were individually transformed into *S. cerevisiae* (strain YPH499) using the lithium acetate method (Gietz and Schiestl, 2007). To express recombinant protein, transformants were selected by 2 days incubation at 30 C on synthetic dextrose (SD) medium lacking uracil and then washed with Milli-Q water. Transformants were resuspended in synthetic complete medium (SG) without uracil containing 2% galactose induction and 4 μg/mL hemin (Misra et al., 2014; Sandeep et al., 2019). After 2 days of incubation at 30 C for protein induction, cells were harvested by centrifugation, crushed with liquid nitrogen with a mortar and pestle, and then resuspended in lysis buffer as previously described (Misra et al., 2014). Western blot analysis was used to confirm the expression of CYP2J2 variants and quantify their protein concentrations with an anti-CYP2J2 antibody.

**TABLE 1:** Primers for *S. cerevisiae* expression vector construction.

Primers	*Sequence (5′–3′)	Amplicon	Variant	Note
2J2-UF	*AAG​TGA​ATT​CAT​GCT​CGC​GGC​GAT​GGG​C*	1,529	wild-type	introducing a EcoRI site
2J2-UR	ACT​C*AGA​TCT*TTA​CAC​CTG​AGG​AAC​AGC​GCA			introducing a Bglll site
2J2-V15A-F	ATT​C*GAA​TTC*ATG​CTC​GCG​GCG​ATG​GGC​TCT​CTG​GCG​GCT​GCC​CTC​TGG​GCA​GcG​GTC	1,529	V15A	introducing a EcoRI site, paired with UR
2J2-R19W-F	ATT​C*GAA​TTC*ATG​CTC​GCG​GCG​ATG​GGC​TCT​CTG​GCG​GCT​GCC​CTC​TGG​GCA​GTG​GTC​GGT​CCA​TCC​TtG​GA	1,529	R19W	introducing a EcoRI site, paired with UR
2J2-G24R-R	GACAGTGCgCAGTAGGA	88	G24R	paired with UF
2J2-G24R-F	TCCTACTGcGCACTGTC	1,458		paired with UR
2J2-V68A-R	ACAGCTGAgCCTCCAGGT	221	V68A	paired with UF
2J2-V68A-F	ACCTGGAGGcTCAGCTGT	1,326		paired with UR
2J2-L166F-R	CTTCAGTGAaGTGTTGGG	515	L166F	paired with UF
2J2-L166F-F	CCCAACACtTCACTGAAG	1,032		paired with UR
2J2-I170T-R	CCTCTTTTgTTGCTTCAG	527	I170T	paired with UF
2J2-I170T-F	CTGAAGCAAcAAAAGAGG	1,020		paired with UR
2J2-173del-R	GCT​GTC​CGT​TCT​CTT​TTA​TT	536	173_173del	paired with UF
2J2-173del-F	AAT​AAA​AGA​GAA​CGG​ACA​GC	1,010		paired with UR
2J2-R200C-R	CTCAAAGCaTTCTCCGA	616	R200C	paired with UF
2J2-R200C-F	TCGGAGAAtGCTTTGAG	930		paired with UR
2J2-K267fs-R	TTC​TGC​AGG​ATT​TCT​GTG​TTT​G	819	K267fs	paired with UF
2J2-K267fs-F	CAA​ACA​CAG​AAA​TCC​TGC​AGA​A	721		paired with UR
2J2-H297R-R	GGTTTTCTTCAcGGAAAC	911	H297R	paired with UF
2J2-H297R-F	GTTTCCgTGAAGAAAACC	636		paired with UR
2J2-G312R-R	CTCGGTTCtGGCAAAGA	952	*8	paired with UF
2J2-G312R-F	TCTTTGCCaGAACCGAG	594		paired with UR
2J2-A355T-R	ACTCCCGGGtGGCTGTG	1,082	A355T	paired with UF
2J2-A355T-F	CACAGCCaCCCGGGAGT	464		paired with UR
2J2-N374I-R	GGATGATGaTGCCCATTC	1,139	N374I	paired with UF
2J2-N374I-F	GAATGGGCAtCATCATCC	408		paired with UR
2J2-A391T-R	GTACCCAGtCAAAGTGG	1,189	A391T	paired with UF
2J2-A391T-F	CCACTTTGaCTGGGTAC	357		paired with UR
2J2-R446W-R	GCATGCCCaCTTTCCTA	1,354	R446W	paired with UF
2J2-R446W-F	TAGGAAAGtGGGCATGC	192		paired with UR
2J2-T470I-R	GGGCCTGAAGaTAAATT	1,429	T470I	paired with UF
2J2-T470I-F	AATTTAtCTTCAGGCCC	117		paired with UR

*The mutated site of each variant is illustrated as lower-case letters. Introduced EcoRI and BglII restriction sites are illustrated as italic fonts.

### 2.5 Evaluation of CYP2J2-mediated drug metabolic activity

To better understand the drug catalytic characteristics of the newly identified CYP2J2 variants, two distinct CYP2J2-specific probe substrates, ebastine and terfenadine, were included in the *in vitro* drug metabolic activity evaluation experiment, as previously described (Lee et al., 2015; Jeong et al., 2018). In brief, the incubation mixtures contained CYP2J2 yeast lysate (0.1–0.5 pmol of CYP2J2 protein), 0.2–1 pmol of cytochrome b5 (CYP2J2/b5 = 1:2), 100 mM potassium phosphate buffer (pH 7.4) and a series of different concentrations of substrates (0.05, 0.1, 0.25, 0.5, 1, 2.5 and 5 μM for ebastine and 0.25, 0.5, 1, 2.5, 5, 10 and 20 μM for terfenadine). After preincubation with yeast microsomes in a volume of 95 μL for 5 min at 37°C, a 1 mM NADPH regenerating system was added to start the reaction in a final volume of 100 μL, and the hydroxylation reactions were incubated at 37 C in a thermoshaker. The reaction proceeded for 20 min and was terminated by the addition of cold acetonitrile (100 μL for ebastine and 200 μL for terfenadine) containing the internal standard (midazolam, 200 ng/mL). The protein was discarded by centrifugation (12,000× g, 15 min), and aliquots of the supernatant were analyzed by a liquid chromatography-tandem mass spectrometry system.

### 2.6 Analysis method

Chromatographic separation was carried out using the Acquity UPLC system (Waters Corp., United States) and ACQUITYUPLC HSS T3 column (2.1 × 100 mm, 1.8 μm) at 40 C. The mobile phase comprising acetonitrile (A) and 0.1% formic acid water (B) was set with a flow rate of 0.40 mL/min and an injection volume of 5 μL. The gradient program was conducted as follows: 0–0.5 min, 30% A; 0.5–1 min, 30%–95% A; 1–2.5 min, 95% A; 2.5–2.6 min, 95%–30% A; 2.6–3 min, 30% A. The total run time for analysis was 3 min. After each injection, the sample manager underwent a strong wash (methanol-water, 50/50, V/V) and a weak wash (methanol-water, 10/90, V/V). The XEVO TQD triple quadrupole mass spectrometer was equipped with electrospray ionization (ESI), and multiple reaction monitoring (MRM) mode was selected for quantization. The precursor ion and product ion were m/z 486.20→167.00 (Cone 50 V, Collision 28 V) for hydroxyebastine, m/z 488.29→452.25 (Cone 48 V, Collision 28 V) for terfenadine alcohol and m/z 325.98→291.07 (Cone 50 V, Collision 26 V) for midazolam (internal standard, IS). Masslynx 4.1 software (Waters Corp., United States) was applied for data acquisition.

### 2.7 Data analysis and statistics

As previously reported, the drug catalytic activities of the CYP2J2.1 and CYP2J2 variants were estimated with two CYP2J2 probe substrates: ebastine and terfenadine (Dai et al., 2013; Chen et al., 2020). After normalizing the CYP enzyme amount in each reaction, the enzymatic kinetic parameters Michaelis constant (*K*_*m*_), maximum reaction velocity (*V*_max_) and intrinsic clearance (Clint, *V*_max_/K_m_) were calculated by Michaelis‒Menten analysis using GraphPad Prism (version 8; GraphPad Software, United States). Kinetic data for each variant are presented as the mean ± S.D. of three microsomal preparations. Statistical analyses were performed in SPSS software (version 16.0, United States) using one-way ANOVA and Dunnett’s test to estimate the significant differences in the catalytic activities between CYP2J2.1 and other variants; *p* < 0.05 was considered statistically significant.

## 3 Results

### 3.1 Investigation of CYP2J2 genetic polymorphisms in the Chinese Han population

**FIGURE 2:**
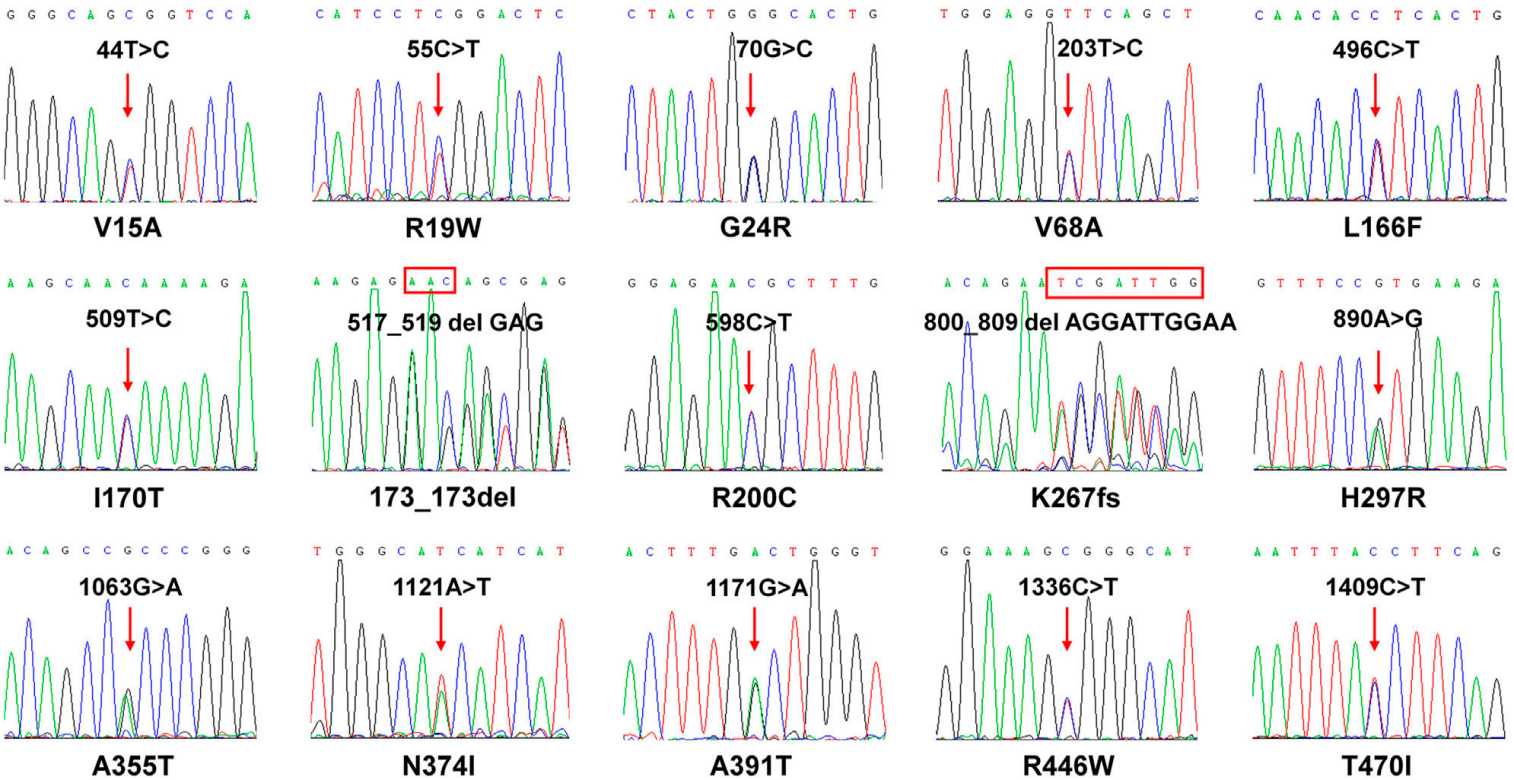
Sequencing results of *CYP2J2* in subjects carrying 15 *CYP2J2* variants. Sanger sequencing results of 15 heterozygous *CYP2J2* variants are shown. The red arrow indicates that the *CYP2J2* variant was detected in the relative position of the coding region. The rectangular box shows the amino acid substitution at the codon. The amino acid alteration is shown below each sequencing result.

To investigate the genetic characteristics of the *CYP2J2* gene in the Chinese Han population, a second-generation sequencing method based on genotyping for the *CYP2J2* gene was performed in 1,163 unrelated healthy individuals of Chinese Han who were recruited in the Department of Cardiology of Beijing Hospital. This multiplex PCR amplicon sequencing method aimed to find the genetic variations in the promoter and exon regions of the *CYP2J2* gene in a time-saving and cost-effective manner (Figure 1). Then, the identified variations in the promoter and nonsynonymous variants in exons were further validated by Sanger sequencing technology. Except for CYP2J2*1, the allelic variants CYP2J2*7 and CYP2J2*8 showed the highest allele frequencies, with frequencies of 4.69% and 0.09%, respectively (Tables 2, 3). Unexpectedly, a high polymorphic status of *CYP2J2* was detected in the Chinese Han population, in which a total of 13 other variation sites in the promoter region (Table 3; Supplementary Figure S1) and 15 nonsynonymous variants in exons (Figure 2; Table 2) were discovered in this study, although they have not been named new alleles by the PharmVar Consortium (Tables 2, 3) (Gaedigk et al., 2018). As illustrated in Figure 2; Table 4, carriers with 15 *CYP2J2* variants were all heterozygous, and these sequence variants could result in an amino acid substitution, deletion or frame shift effect at the protein level. The allelic frequencies of these variants were all below 1% (Table 2). Moreover, 5 genetic variations in the promoter region and 5 nonsynonymous variants (V15A, G24R, V68A, L166F, and A391T) were novel and had not been designated with any RS number in the public SNP database (Tables 2, 3) (Karczewski et al., 2020). For variants in the promoter region, −998A>G, −587G>A, −552C>G and CYP2J2*7 (−76G>T) showed higher allele frequencies, and −998A>G was found to be the most prevalent *CYP2J2* promoter variant with an allele frequency of 45.79% (Table 3). Haplotype analysis also revealed that CYP2J2*7 was commonly linked with the other two variation sites −587G>A and −552C>G (Table 5).

**TABLE 2:** Allelic frequencies of nonsynonymous *CYP2J2* variations discovered in 1,163 Chinese Han subjects.

Chr: genomic position	cDNA change	Amino-acid effect	rsID	Region	Allele	Subject number (*n*)	Allele frequency (%)
1:59926703	44T>C	V15A*	—	Exon 1	Novel	1	0.04
1:59926692	55C>T	R19W	rs181737961	Exon 1		1	0.04
1:59926677	70G>C	G24R*	—	Exon 1	Novel	1	0.04
1:59926544	203T>C	V68A*	—	Exon 1	Novel	1	0.04
1:59912189	496C>T	L166F*	—	Exon 3	Novel	1	0.04
1:59912176	509T>C	I170T	rs557770949	Exon 3		1	0.04
1:59912165	517_519 del GAG	173_173del	rs757528200	Exon 3		5	0.21
1:59911694	598C>T	R200C	rs201070738	Exon 4		1	0.04
1:59909835	800_809 del AGGATTGGAA	K267fs	rs568437594	Exon 5		3	0.13
1:59907899	890A>G	H297R	rs754057670	Exon 6		1	0.04
1:59907855	934G>A	G312R	rs150461093	Exon 6	CYP2J2*8	2	0.09
1:59904999	1063G>A	A355T	rs144856672	Exon 7		2	0.09
1:59904941	1121A>T	N374I	rs199717190	Exon 7		3	0.13
1:59904891	1171G>A	A391T*	—	Exon 7	Novel	1	0.04
1:59893824	1336C>T	R446W	rs201379188	Exon 9		1	0.04
1:59893751	1409C>T	T470I	rs759510111	Exon 9		1	0.04

*The GenBank accession numbers of the novel *CYP2J2* variations were OQ434051 (V15A), OQ434052 (G24R), OQ434053 (V68A), OQ434054 (L166F) and OQ434055 (A391T), that were deposited into GenBank (https://www.ncbi.nlm.nih.gov/nuccore/).

**TABLE 3:** Variations in the promoter of *CYP2J2* in 1,163 Chinese Han subjects.

Variation	rsID	Subject number (*n*)	Allele frequency (%)
−998A>G	rs7533359	822	45.79
−987G>A	rs918965613	1	0.04
−957C>T*	—	1	0.04
−929G>T	rs368118763	1	0.04
−690G>T	rs141350998	1	0.04
−645del C	rs138763360	1	0.04
−587G>A	rs10889162	194	8.99
−552C>G	rs11572188	105	4.64
−538G>T	rs561602119	1	0.04
−351A>G*	—	1	0.04
−273G>C*	—	1	0.04
−265T>C*	—	1	0.04
−259C>T*	—	1	0.04
−76G>T (CYP2J2*7)	rs890293	105	4.69

The gene position is according to the reference sequence NC_000001.11 in GenBank, and the A of the ATG, translation initiation codon is denoted as nucleotide 1. *The GenBank accession numbers of the variations in the promoter region were OQ434050 (−957C>T), OQ434049 (−351A>G), OQ434048 (−273G>C), OQ434047 (−265T>C) and OQ434046 (−259C>T), that were deposited into GenBank (https://www.ncbi.nlm.nih.gov/nuccore/).

**TABLE 4:** Genotype frequencies of nonsynonymous *CYP2J2* allelic variants in 1,163 Chinese Han subjects.

Amino-acid effect	Genotype	Subject number (*n*)	Frequency (%)
V15A	T/C	1	0.09
R19W	C/T	1	0.09
G24R	G/C	1	0.09
V68A	T/C	1	0.09
L166F	C/T	1	0.09
I170T	T/C	1	0.09
173_173del	GGAG/G	5	0.43
R200C	C/T	1	0.09
K267fs	AAGGATTGGAA/A	3	0.26
H297R	A/G	1	0.09
G312R	*1/*8	2	0.17
A355T	G/A	2	0.17
N374I	A/T	3	0.26
A391T	G/A	1	0.09
R446W	C/T	1	0.09
T470I	C/T	1	0.09

**TABLE 5:** Genetic distribution of main variants in the *CYP2J2* promoter region.

Variation	Subject number (*n*)	Frequency (%)	Heterozygote number	Heterozygote frequency (%)	Homozygote number	Homozygote frequency (%)
−998A>G	733	63.03	490	42.13	243	20.89
−587G>A	46	3.96	42	3.61	4	0.34
−998A>G, −587G>A	43	3.70	43	3.70	0	0.00
−587G>A, −552C>G, −76G>T (CYP2J2*7)	59	5.07	56	4.82	3	0.26
−998A>G, −587G>A, −552C>G, −76G>T (CYP2J2*7)	46	3.96	45	3.87	1	0.09

The gene position is according to the reference sequence NC_000001.11 in GenBank, and the A of the ATG, translation initiation codon is denoted as nucleotide 1.

### 3.2 Expression of wild-type CYP2J2 and variants in *S. cerevisiae*

**FIGURE 3:**
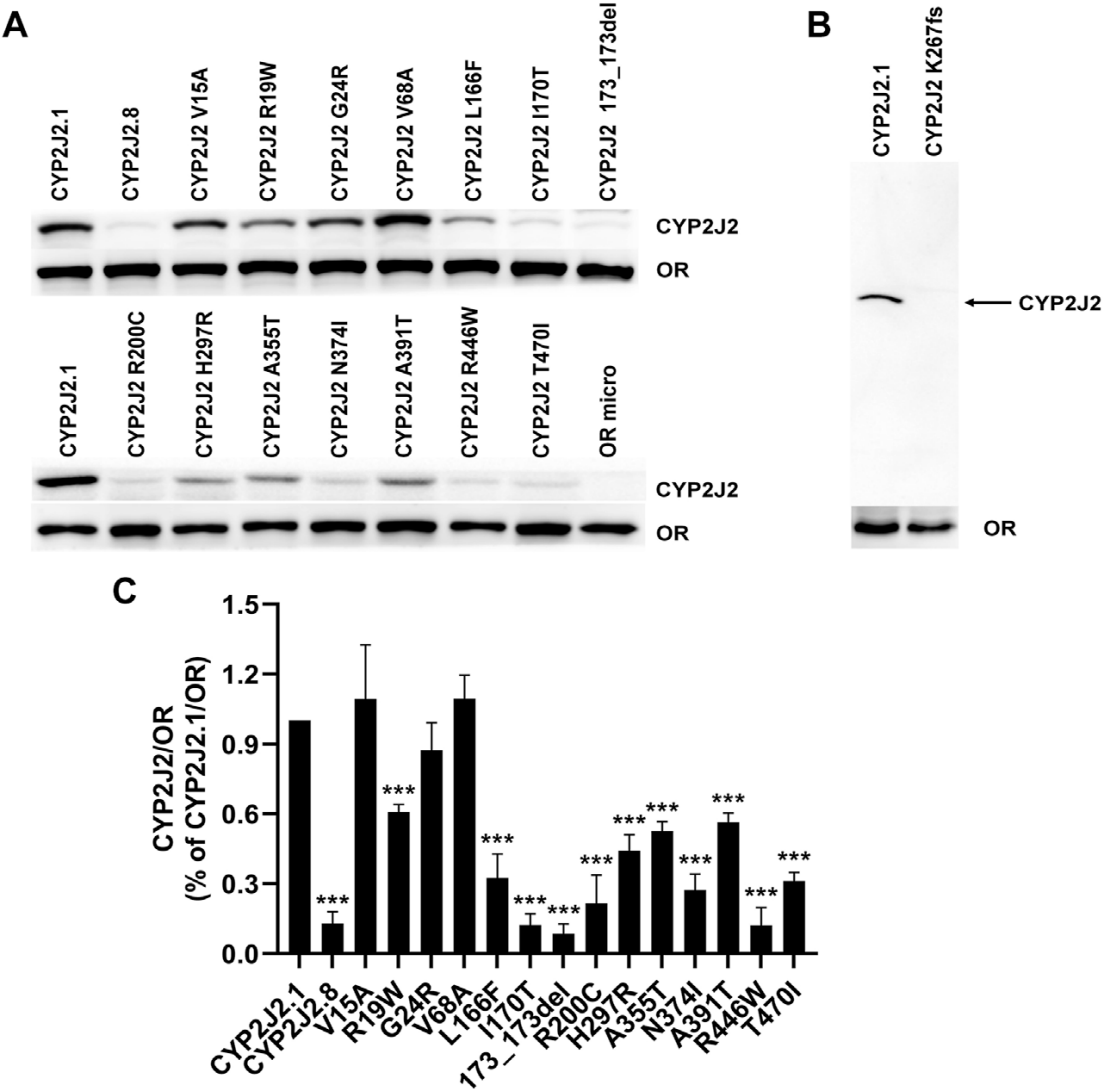
Expression of CYP2J2.1, CYP2J2.8 and 15 variants in *S.* ***cerevisiae*. (A,B)** Western blot analysis of exogenously expressed P450 reductase (OR) and CYP2J2 variants in *S. cerevisiae* cells. **(B)** Immunoblotting results for the frameshift mutation K267fs. OR micro: *S. cerevisiae* cells expressing only the OR enzyme. **(C)** Relative CYP2J2/OR intensities. Each bar represents the mean ± S.D. of three independently performed experiments. *p < 0.05, **p < 0.01, ***p < 0.001 vs*.* CYP2J2.1/OR.

To evaluate the catalytic activities of 15 newly detected CYP2J2 variants, wild-type CYP2J2 enzyme and all variants were recombinantly expressed in *S. cerevisiae*. The expression levels of CYP2J2 proteins were detected with Western blot analysis. As shown in Figure 3, most of the CYP2J2 variants exhibited significantly decreased protein expression relative to that of the wild-type enzyme CYP2J2.1. These variants included CYP2J2.8, R19W, L166F, I170T, 173_173del, R200C, H297R, A355T, N374I, A391T, R446W, and T470I. For variant K267fs, no signals could be detected because the frameshift mutation in this variant could cause the premature termination of translation of CYP2J2 protein in *S. cerevisiae*. The protein levels of other identified CYP2J2 variants, including V15A, G24R and V68A, remained unchanged. These results indicated that different CYP2J2 variants had distinct effects on the protein expression of the CYP2J2 enzyme *in vitro*.

### 3.3 Kinetic characterizations of CYP2J2 variants toward ebastine

**FIGURE 4:**
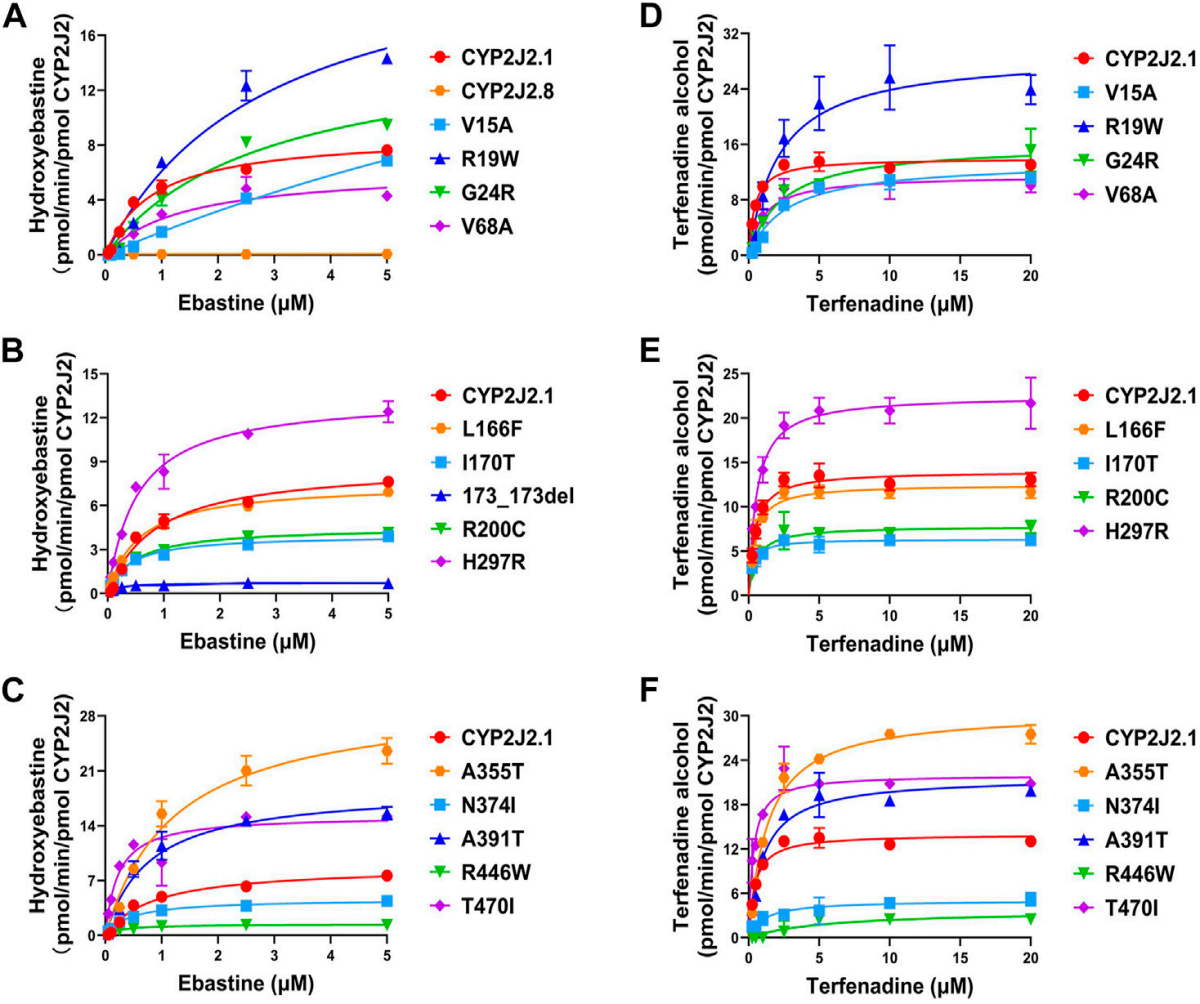
Michaelis‒menten curves of catalytic activity analyses. **(A–C)** Michaelis‒Menten curves of the catalytic activities of recombinant CYP2J2.1, CYP2J2.8 and variants toward ebastine. **(D–F)** Michaelis‒Menten curves of CYP2J2 catalytic activities toward terfenadine. Each point represents the mean ± S.D. of three separate experiments.

To identify the potential effects of 15 newly detected CYP2J2 variants on catalytic activities, two typical CYP2J2 probe substrates, ebastine and terfenadine, were used for drug metabolic activity analysis *in vitro*. Except for CYP2J2 K267fs, which lost the catalytic activity of ebastine, CYP2J2.8 and 14 CYP2J2 variants exhibited different *K*_*m*_, *V*_max_ or Clint values (Figure 4; Table 6). Specifically, CYP2J2.8, 173_173del, N374I, and R446W exhibited extremely low catalytic abilities for the metabolism of ebastine. In addition, 8 CYP2J2 variants exhibited markedly decreased *V*_max_, and the remaining 7 variants showed significantly increased *V*_max_ compared with CYP2J2.1. The *K*_*m*_ value was obviously reduced in 9 CYP2J2 variants and markedly increased in 5 variants relative to CYP2J2.1. One CYP2J2 variant exhibited a *K*_*m*_ value comparable to that of CYP2J2.1. Overall, CYP2J2.8 and six of the 14 newly detected variants (V15A, R19W, G24R, V68A, 173_173del, and R446W) had Clint values that were lower than that of CYP2J2.1. However, 5 variants (L166F, H297R, A355T, A391T and T470I) showed increased Clint values relative to that of the wild-type enzyme. The other 3 variants had no significant differences in the Clint value of ebastine hydroxylation compared with CYP2J2.1.

**TABLE 6:** Kinetic parameters for catalytic activities of recombinant CYP2J2 wild-type and variants against ebastine.

Substrate	Allelic protein	*V*_max_ (pmol/min/pmol)	*K*_*m*_ *(μM)*	Clearance (*V*_max_/K_m_)	Relative clearance (/CYP2J2.1, %)
Ebastine	T470I	15.31 ± 0.76***	0.22 ± 0.01***	68.66 ± 6.25***	662.27***
	H297R	13.45 ± 0.88***	0.53 ± 0.04***	25.52 ± 1.87***	246.12***
	A355T	30.46 ± 0.81***	1.23 ± 0.09**	24.83 ± 1.57***	239.5***
	A391T	18.99 ± 0.91***	0.81 ± 0.2	24.37 ± 4.99**	235.03**
	L166F	7.52 ± 0.34**	0.55 ± 0.06**	13.66 ± 0.79**	131.78**
	I170T	3.98 ± 0.03***	0.38 ± 0.01***	10.53 ± 0.26	101.57
	N374I	4.63 ± 0.14***	0.45 ± 0.05***	10.4 ± 0.77	100.33
	CYP2J2.1	8.82 ± 0.09	0.85 ± 0.05	10.37 ± 0.55	100
	R200C	4.55 ± 0.21***	0.48 ± 0.07**	9.56 ± 1.1	92.21
	R19W	23.93 ± 1.27***	2.94 ± 0.37***	8.2 ± 0.66*	79.06*
	G24R	15.64 ± 1.23***	2.87 ± 0.56**	5.53 ± 0.63***	53.38***
	R446W	1.44 ± 0.08***	0.28 ± 0.05***	5.21 ± 0.59***	50.27***
	V68A	6.27 ± 0.35***	1.41 ± 0.14**	4.49 ± 0.63***	43.35***
	173_173del	0.73 ± 0.07***	0.17 ± 0.05***	4.42 ± 0.88***	42.66***
	V15A	41.25 ± 18.62*	24.81 ± 13.59*	1.78 ± 0.33***	17.19***
	CYP2J2.8	0.06 ± 0.01***	0.24 ± 0.17**	0.31 ± 0.13***	2.97***
	K267fs	NA	NA	NA	NA

Data are presented as the mean ± S.D., of 3 different experiments. *p < 0.05, **p < 0.01, ***p < 0.001 vs*.* CYP2J2.1; NA, not applicable.

### 3.4 Kinetic characterizations of CYP2J2 variants toward terfenadine

As shown in Table 7, the CYP2J2.8 variant completely lost its enzymatic activity for terfenadine hydroxylation, as previously described (Jeong et al., 2018). Due to the loss or weak expression of the enzyme, variant K267fs or 173_173del showed undetectable catalytic activities toward terfenadine. Similar to the results of ebastine, N374I and R446W only produced low concentrations of terfenadine alcohol after the reaction. Moreover, 13 CYP2J2 variants exhibited changed *V*_max_ or *K*_*m*_ values compared with the values of CYP2J2.1. Specifically, four variants (L166F, I170T, H297R and A355T) exhibited similar Clint values to that of wild-type CYP2J2.1; one variant, T470I, had a higher Clint value than that of CYP2J2.1; the other 8 variants (V15A, R19W, G24R, V68A, R200C, N374I, A391T and R446W) showed significantly lower Clint than CYP2J2.1 (Figure 4; Table 7).

**TABLE 7:** Kinetic parameters for catalytic activities of recombinant CYP2J2 wild-type and variants against terfenadine.

Substrate	Allelic protein	*V*_max_ (pmol/min/pmol)	*K*_*m*_ *(μM)*	Clearance (*V*_max_ */*K_m_)	Relative clearance (/CYP2J2.1, %)
Terfenadine	T470I	21.95 ± 0.86***	0.31 ± 0.06	71.89 ± 13.84*	223.75*
	H297R	22.64 ± 1.31***	0.58 ± 0.16	40.7 ± 7.78	126.66
	CYP2J2.1	14.02 ± 0.44	0.45 ± 0.09	32.13 ± 5.94	100
	L166F	12.5 ± 0.21**	0.43 ± 0.04	29.3 ± 3.18	91.18
	I170T	6.37 ± 0.28***	0.28 ± 0.09	24.36 ± 6.13	75.83
	A355T	30.62 ± 0.62***	1.35 ± 0.05***	22.78 ± 0.87	70.91
	A391T	21.72 ± 1.1***	1.03 ± 0.1**	21.26 ± 1.06*	66.15*
	R200C	7.81 ± 0.6***	0.53 ± 0.04	14.71 ± 0.78**	45.78**
	R19W	29.08 ± 3.29**	2.3 ± 0.73*	13.54 ± 4.43*	42.14*
	V68A	11.58 ± 0.67**	1.13 ± 0.32*	10.65 ± 2.14**	33.13**
	G24R	16.44 ± 3.43	2.59 ± 1.17*	6.82 ± 1.55**	21.23**
	V15A	13.64 ± 0.49	2.82 ± 0.53**	4.93 ± 0.72**	15.35**
	N374I	5.13 ± 0.66***	1.2 ± 0.4*	4.63 ± 1.71**	14.4**
	R446W	3.59 ± 0.54***	5.29 ± 3.81*	0.87 ± 0.53**	1.8**
	173_173del	NA	NA	NA	NA
	CYP2J2.8	NA	NA	NA	NA
	K267fs	NA	NA	NA	NA

Data are presented as the mean ± S.D., of 3 different experiments. *p < 0.05, **p < 0.01, ***p < 0.001 vs*.* CYP2J2.1; NA, not applicable.

### 3.5 The catalytic characteristics of CYP2J2.1 and variants between ebastine and terfenadine

**FIGURE 5:**
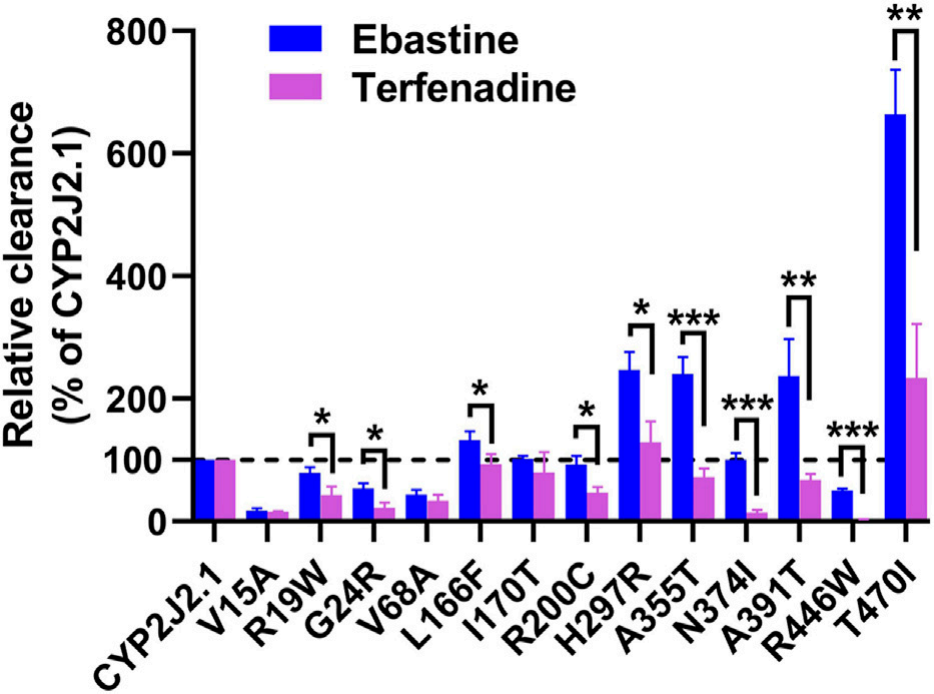
The relative clearance rates of ebastine and terfenadine among CYP2J2.1 and variants. Relative clearance rates of 13 CYP2J2 variants between ebastine and terfenadine were compared, except CYP2J2.8, 173_173del and K267fs. *p < 0.05, **p < 0.01, ***p < 0.001 between ebastine and terfenadine (*n* = 3).

To better understand the selective properties of *CYP2J2* genetic polymorphisms on drug metabolism, the relative clearance Clint values of variants between ebastine and terfenadine were compared in this study. As shown in Figure 5, most of the CYP2J2 variants showed similar patterns for the relative enzymatic activities for two histamine H1 receptor antagonist drugs, but some specific variants still exhibited substrate-dependent activities for them, which was in accordance with the substrate-dependent manner of some other CYP proteins (Han et al., 2021; Parikh et al., 2021). In brief, 3 variants (CYP2J2.8, 173_173del and K267fs) had undetectable or relatively low catalytic activities for both ebastine and terfenadine. Five variants, including V15A, R19W, G24R, V68A and R446W, caused a decline in Clint values for both substrates (Figure 5; Tables 6, 7). Only variant T470I exhibited a significant elevation in Clints for both substrates. In addition, one variant, I170T, showed unchanged Clints for two substrates relative to that of the wild-type. Additionally, L166F, R200C, H297R, A355T, N374I and A391T showed different patterns for the metabolism of the two substrates relative to that of the wild-type enzyme. Specifically, variant A391T exhibited higher metabolic activity toward ebastine but showed significantly decreased activity for the metabolism of terfenadine. Overall, all the identified CYP2J2 variants exhibited higher relative clearance values toward ebastine than toward terfenadine.

## 4 Discussion

Similar to other CYP subfamily members, *CYP2J2* exhibits distinct genetic polymorphisms and allelic frequencies among different races and individuals. However, few studies have focused on the *CYP2J2* polymorphism, and limited *CYP2J2* polymorphic sites have been identified and assigned, especially in the Chinese population (Wang et al., 2006; Liu et al., 2007; Zhang et al., 2016a; Zhang et al., 2016b; Li et al., 2021). In this study, we performed large-scale genetic screening by sequencing the promoter and exon regions of the *CYP2J2* gene in 1,163 randomly recruited healthy Chinese Han individuals and made systematic functional predictions of CYP2J2 nonsynonymous variants for drug metabolism *in vitro*. In total, two previously reported alleles (CYP2J2*7 and CYP2J2*8), 13 other *CYP2J2* promoter variants and 15 unassigned *CYP2J2* nonsynonymous variants were identified. Most *CYP2J2* promoter variants and nonsynonymous variants were not identified in previous studies of Chinese Han individuals, and five of 15 *CYP2J2* nonsynonymous variants were novel (Lee et al., 2005; Karczewski et al., 2020; Li et al., 2021). The identified allele frequencies of CYP2J2*7 and CYP2J2*8 were 4.69% and 0.09%, respectively, which were equal to those in previous studies (Li et al., 2021). Except for CYP2J2*7, three promoter variants (including −998A>G, −587G>A, −552C>G) showed high allele frequencies in Chinese Han individuals, and −552C>G, −587G>A and CYP2J2*7 were commonly carried by the same subjects. Previous studies confirmed that the promoter variant CYP2J2*7 was the typical defective allele and decreased epoxygenase activity *in vivo* (Murray, 2016). However, the functions of other identified promoter variants remain to be undetermined. Although our study showed that the allelic distributions of 15 *CYP2J2* nonsynonymous variants were rare in Chinese Han individuals, the results revealed that the *CYP2J2* genetic polymorphisms in Chinese Han individuals were far beyond previous identification. To date, CYP2J2*1, CYP2J2*7, *8, *9, M116V, V188I, N190S, R200C, Y203C and A355T have been reported in Chinese Uyghur, Chinese Wa, Chinese Tibetan or South Asian. In this study, the allelic frequencies of R200C and A355T in Chinese Han population were 0.04% and 0.09% respectively. Whereas they were reported to be 0.5% and 1% in Chinese Uyghur individuals. Moreover, CYP2J2*7 showed allelic frequencies of 4.69%, 3.5%, 4.5%, 12%, 3.4%, 6.2%, and 4.2% in our study, Chinese Uygur, Chinese Zhuang, Taiwanese, Mongolian, Japanese and Korean, respectively (Zhang et al., 2016b; Cao et al., 2018; Li et al., 2021), and CYP2J2*8 were only reported in the Chinese Uygur, Korean, and our study, with the allelic frequencies of 0.5%, 0.8%, and 0.09%, respectively (Li et al., 2021). Therefore, the regional and ethnic distribution pattern of CYP2J2 allelic variants were quite different among distinct Asian populations.

In the *in vitro* functional evaluation experiments, we found that the expression of CYP2J2 (0.031 pmol/μg) was relatively lower than that of CYP2C9 (0.17 pmol/μg) in the *S. cerevisiae* expression system, the tendency of which was equal to the protein levels of CYP isoform expression in the liver (Zhao et al., 2021). However, CYP2J2 is predominantly involved in the metabolism of approximately 3% of drugs in the clinic, and China has a large population of more than 1.4 billion. The functional characteristics of these alleles are still necessary and valuable for clinical practice. Previous studies confirmed that the genetic variations of CYP2J2 exerted distinct catalysis activities for substrates. CYP2J2.2, CYP2J2.3, CYP2J2.4, and CYP2J2.6 variations showed decreased metabolic activities toward both arachidonic acid and linoleic acid, and CYP2J2*10 encoded a reduced-function protein (King et al., 2002; Lee et al., 2005; Gaedigk et al., 2006; Berlin et al., 2011). The enzymatic activities of the CYP2J2.5 and CYP2J2.9 variants were comparable to that of CYP2J2.1. Using the Sf9 cell system, a previous study found that CYP2J2.8 almost completely lost the catalytic activity of ebastine (Lee et al., 2005). With *Escherichia coli* expression system, CYP2J2.8 was proven to have defective expression of the CYP2J2 holoenzyme and showed no CYP2J2-mediated metabolism of terfenadine (Jeong et al., 2018). We also demonstrated that CYP2J2.8 showed much lower protein expression than CYP2J2.1, nearly lost the ebastine catalytic activity and exhibited undetectable catalytic activity of terfenadine in the *S. cerevisiae* expression system. Regarding the immunoblotting results and Clint values of ebastine and terfenadine in this study, most of the 15 CYP2J2 variants markedly decreased the protein expression and catalytic activities for substrates, especially the variants with higher allele frequencies, including CYP2J2.8, 173_173del, K267fs and R446W. For variants with low protein expression, most of them exhibited significantly decreased metabolic activity toward tested probe drugs. For example, no activity could be detected for variant K267fs toward probe drugs ebastine and terfenadine. Additionally, variants 173_173del and CYP2J2.8 showed no metabolic activity for terfenadine and extremely low activity for another probe drug ebastine. We believe that less expression of these 3 variants, caused by protein instability or other factors, may be the main reason for their extremely lower catalytic activities. However, this situation is not applicable to all variants with lower protein expression, such as variants I170T, R200C, N374I and T470I. As illustrated in Figures 3, 4, I170T, R200C and N374I exhibited similar catalytic activity toward ebastine to that of wild-type enzyme although they showed significantly decreased protein expression level compared to the wild type. Additionally, variant T470I exhibited significantly increased metabolic ability toward both ebastine and terfenadine (Figures 4, 5). These data indicated that some CYP2J2 variants with abolished protein expression may exhibit increased or unchanged catalytic activities, phenomena that were also found in other CYP variants (Amorosi et al., 2021). Moreover, the protein expression manner and level vary in distinct protein expression system and might be different between in yeast and in human (Durocher et al., 2002; Martinez et al., 2012). Therefore, the catalytic functions of these CYP2J2 variants *in vivo* need to be further evaluated in consideration of the complicated factors of individual differences. Furthermore, our results revealed that all 15 detected CYP2J2 variants displayed higher relative clearance of ebastine than that of terfenadine, which was the same as the results of CYP2J2.8 metabolizing ebastine and terfenadine. This result highlighted that CYP2J2 variants tended to strongly influence ebastine hydroxylation compared to terfenadine hydroxylation. This finding might be related to the different chemical construction and substrate binding affinities. The exact underlying mechanisms need further in-depth study. Furthermore, CYP2J2 is the highest expression CYP in human cardiomyocytes and oxidizes arachidonic acid to cardioprotective regioisomers of cis-epoxyeicosatrienoic acids *in vivo* (Kim et al., 2022). Previous studies revealed that CYP2J2 plays an important role in the cardiovascular system and that *CYP2J2* genetic polymorphisms are associated with the risk of cardiac disease (Valencia et al., 2022). Cardiac-specific CYP2J2 overexpression had beneficial effects on cardiovascular disease progression and recovery, such as improving the outcomes of ischemia and/or ischemia‒reperfusion injuries, decreasing arrhythmia of hypertension and delaying hypertension development (Xiao et al., 2010; Islam et al., 2017; Aliwarga et al., 2018). Furthermore, it was confirmed that the typical defective allele CYP2J2*7 was an independent risk factor for premature myocardial infarction in the Chinese Han population and increased the risk of MI in a predominantly Caucasian population and South Indian population (Liu et al., 2007; Marciante et al., 2008; Arun Kumar et al., 2015). CYP2J2*7 was also associated with an elevated risk of ischemic stroke in Chinese Han individuals and increased the risk of atherosclerosis, hypertension, and coronary artery disease in many ethnic groups (Spiecker et al., 2004; Li et al., 2015; Aliwarga et al., 2018). Moreover, previous study confirmed TT homozygote carriers of *CYP2J2* gene rs1155002 is susceptibility to essential hypertension, especially in females, and had higher systolic blood pressure in the Chinese Han population (Wu et al., 2007). In this study, the drug metabolic activity analysis experiment revealed that most of detected CYP2J2 variants exhibited significantly decreased catalytic activities for probe drugs ebastine and terfenadine, although some of variants showed increased activities for the metabolism of typical CYP2J2 substrates (Tables 6, 7). Our data indicated that newly detected CYP2J2 variants might have impacts to the metabolism of clinically used CYP2J2-mediated drugs or influence the development of several diseases, especially cardiac disease, especially for the carriers with mutations that showed both extremely low protein expression ability and low metabolic activity. Thus, getting the genetic information of CYP2J2 might be relevant to the personalized medicine in Chinese populations when taking CYP2J2-mediated drugs or beneficial to the prevention for CYP2J2 related disease development. However, these speculations need further and systematic investigation.

In summary, this study discovered 15 unassigned *CYP2J2* nonsynonymous variants in a large Chinese Han population and systematically assessed their catalytic activities using the *S. cerevisiae* expression system. Most of the identified variants, especially the variants with relatively high allele frequencies, significantly decreased catalytic activities toward the metabolism of ebastine and terfenadine. These findings expand our understanding of the functional effects of *CYP2J2* genetic polymorphisms and provide some foundation for future clinical studies regarding individual variation in CYP2J2-mediated drug efficacy, adverse drug reactions and CYP2J2-related disease. However, whether subjects carrying these variant alleles show differences in CYP2J2-related drug efficacy or require a higher dose to maintain drug effects must be confirmed by further *in vivo* studies.

## Data Availability

The datasets presented in this study can be found in online repositories. The names of the repository/repositories and accession number(s) can be found below: NCBI GenBank [https://www.ncbi.nlm.nih.gov/nuccore/], OQ434046-OQ434055.
