# Supplementary material for: Characterization of 15 CYP2J2 variants identified in the Chinese Han population on the metabolism of ebastine and terfenadine in vitro
Source: Front Pharmacol. 2023 May 23;14:1186824. doi: 10.3389/fphar.2023.1186824 (PMC10242136; doi:10.3389/fphar.2023.1186824)
Supplement: Supplementary file 1 [file Table1.DOCX]

Supplementary Material

**Characterization of 15 CYP2J2 variants identified in the Chinese Han population on the metabolism of** **ebastine and terfenadine *in vitro***

**Li-li Zou^1†^, Fang-ling Zhao^1†^, Yu-ying Qi^1^, Shuang-hu Wang^2^, Quan Zhou^2^, Pei-wu Geng^2^, Yun-fang Zhou^2^, Qing Zhang^3^, Hao Chen^3^, Da-peng Dai^1*^, Jian-ping Cai^1*^, Fu-sui Ji^1, 3*^**

**^*^Corresponding author:**

Da-peng Dai, [daidapeng@163.com](mailto:daidapeng@163.com), Jian-ping Cai, caijp61@vip.sina.com, Fu-sui Ji, [jfs_bjh@163.com](mailto:jfs_bjh@163.com)

**Table S1.** Primers used for multiplex PCR amplicon sequencing of the human *CYP2J2* gene.

| ^*^Forward primer (5’–3’) | ^*^Reverse primer (5’–3’) | Amplicon (bp) | Position | Region |
| --- | --- | --- | --- | --- |
| ACCAGCCTCCTATTAATTGCTTACC | CCAGAATGTCATGTTGCAGAAGC | 244 | chr1:59928577-59928821 | Promoter |
| GGATATCCCTAGACCTCTTAGCCA | GTGGCATTTGAGCCATAACTCAC | 229 | chr1:59928412-59928641 | Promoter |
| GTCTAGAATGGGGTTTCTGTCACG | AGCATGAGAAACCCCACAGAAC | 243 | chr1:59928240-59928483 | Promoter |
| GAACAATTTTCAGGGACTTAAATTATTTTAGTTT | GGCTCTAATTCACTTAATTTAATAGATGGATAGT | 259 | chr1:59928079-59928338 | Promoter |
| TCCACTGATACGGGAATGAGCA | TCCAGAGAGAAATACTAGTCAGAGCT | 259 | chr1:59927902-59928161 | Promoter |
| AGCTGTAGTAAAACAGGAAAATGTGG | CCCGGCTAATTTTTGTATTTTTGGTAC | 243 | chr1:59927730-59927973 | Promoter |
| GATCACCTATGGTCAGGAGATTGA | AGAGTCACATATTCTGCTTACAATATTTTATTT | 258 | chr1:59927556-59927814 | Promoter |
| TCACCTATGGTCAGGAGATTGAGA | CACTCATGTACTCAACATAGAGTCACA | 274 | chr1:59927538-59927812 | Promoter |
| CAGATCACCTATGGTCAGGAGATTG | CACACTCATGTACTCAACATAGAGTCA | 280 | chr1:59927536-59927816 | Promoter |
| AGGTTGCAGTGAGCTGAACTC | CTCTATTAGGCTCCTCTCCAAAGC | 260 | chr1:59927396-59927656 | Promoter |
| GATGGTATGAAAGGCTGGAACCT | GGTGAACAAATAAATCCACCCTTCTG | 242 | chr1:59927221-59927463 | Promoter |
| TCCAGACACTTTCAAATACAGGACC | TGGAACTGTGCCTGGTAAACAG | 258 | chr1:59927030-59927288 | Promoter |
| GGATGTGCTGCCTGGCAT | AGCACCCTGCGAAGATGC | 252 | chr1:59926861-59927113 | Promoter |
| CTAGCCTGGCCTTTTCTGAGAC | AGTAGGAGAGTCCGAGGATGGA | 255 | chr1:59926678-59926933 | Promoter and Exon 1 |
| AGGAGGACGTCTGAGCCAT | AGGACACGCTAGGCACCT | 259 | chr1:59926504-59926763 | Exon 1 |
| CCTTCCTTGGCAACTTCTTCCT | GAAAGTGCAGGTGGAATCATGC | 252 | chr1:59926343-59926595 | Exon 1 |
| ACCAATGTACACTCACTCACACA | GGTTCCCATATTTCTTCACAAACTGA | 184 | chr1:59916078-59916262 | Exon 2 |
| TCTGAACACATGGACATGTGCA | CAACATCAAACACTCACCTTTCGT | 257 | chr1:59915897-59916154 | Exon 2 |
| TGGAGCTTGGTGACATATCTGC | TATGGGGCTGGTTTCTAGGAGT | 243 | chr1:59915826-59916069 | Exon 2 |
| TCTCTGGGCAGTTTTCAAGTGC | ACCTTCTTTGCTCCTTCCATGC | 174 | chr1:59912266-59912440 | Exon 3 |
| TGCTGGATATTCATATTATGGAATCAGAATAC | ATGAACAAATGGGCCACAGTCT | 257 | chr1:59912112-59912369 | Exon 3 |
| AGCTTAGAGGAACGCATTCAGG | ACAGTGCTGGGCATAGAACAAG | 164 | chr1:59912061-59912225 | Exon 3 |
| ACTGAATTTAAGCCCTGGTCCG | GTCAAAAGGCTGTCCTGAAGGT | 221 | chr1:59911754-59911975 | Exon 4 |
| TCCATCAGAAGGATCCATCTTGC | GACATCGGCCCCAATTTACTGA | 252 | chr1:59911556-59911808 | Exon 4 |
| AAGGCAGCTGTCTCTCCATATC | CATAGGTGGATAGGCGTATGTCA | 256 | chr1:59911349-59911605 | Exon 4 |
| AGAGAGAAAAGAGGGGTCAAGGA | TCCAGTTGCTGAAGAGAGTTTGG | 213 | chr1:59909890-59910103 | Exon 5 |
| CCATGTTATCTGCATGATTGTTTTGTTT | TTGTGCTCTAAGAACAAAATACAAAATGAC | 260 | chr1:59909739-59909999 | Exon 5 |
| TGTTTTTCTCAATTAGCTCTACAATGTCTT | CCTCTATTTGTGCTCTAAGAACAAAATACA | 244 | chr1:59909732-59909976 | Exon 5 |
| GTTTTGTTTTTCTCAATTAGCTCTACAATGTC | CCTCTATTTGTGCTCTAAGAACAAAATACAA | 248 | chr1:59909732-59909980 | Exon 5 |
| GGATTGGAATCCTGCAGAAACAAG | ACCTTATCTGTAACCCAGTGAAGC | 241 | chr1:59909603-59909844 | Exon 5 |
| CTTGGGGCAGGACAATGCTAAT | GAAGTAGGATTGCCTGTGTGCT | 210 | chr1:59907907-59908117 | Exon 6 |
| TACGGCAATCAGGAATCCTCTG | GAGGCACTATTCTGTCCTGGTT | 250 | chr1:59907738-59907988 | Exon 6 |
| ATGGGCTCTGCTTTATATGGCC | GGCCCATCACAATTCAGACAGA | 220 | chr1:59907606-59907826 | Exon 6 |
| GACATCTTGGAGACCACTCATACC | GGGATGATGTTGCCCATTCTCT | 208 | chr1:59904931-59905139 | Exon 7 |
| GAACCCTTCCCTCTTTACCAGA | CCCCTAAAAGATGGGCTTGAAG | 245 | chr1:59904834-59905079 | Exon 7 |
| GTACCACCTGCCCAAGGTAATT | CCCACAGGAAAGAAAAGCCAGT | 260 | chr1:59904626-59904886 | Exon 7 |
| TTCCCAGCAGGGAAATCCAATC | GTCAGGATCATGGTACCCTAGAGA | 231 | chr1:59901086-59901317 | Exon 8 |
| GTCAAGCCTTGCCTGAGTCT | CAAGGGAAAACAGGAGAGAGCA | 253 | chr1:59900886-59901139 | Exon 8 |
| TTCGTGTTCCTGGACTTCTGC | ATGCCCGCTTTCCTGTAAGAC | 215 | chr1:59893817-59894032 | Exon 9 |
| ACCTGAGAAGATAACCCGTCTTTT | GTCTTCTTACTTTCCTTGCCCCTT | 257 | chr1:59893611-59893868 | Exon 9 |
| CCAAACAATGAGAAGCTGAGCC | CAACACATCTGAGCAGACACCA | 173 | chr1:59893567-59893740 | Exon 9 |

^*^All primers contain a universal part in the 5’ direction, and only the sequences for the 3’ specific part are listed in this table.

**Table S2.** Primers used for human *CYP2J2* gene amplification or Sanger sequencing.

| PCR primers | | |  |  |
| --- | --- | --- | --- | --- |
| Region | Forward primer (5’–3’) | Reverse primer (5’–3’) | Fragment size (bp) | Annealing temperature (°C) |
| Promoter and Exon 1 | CCTTAGAAAGAGAACAAGAGACTTC | GCTGGAGGGCTCAGTTAGTG | 1794 | 55 |
| Exon 3 to Exon 5 | ATTTCTCTGGGCAGTTTTCAAGTG | GGCTGCGAAAATGGTAGGTC | 3484 | 55 |
| Exon 6 and Exon 7 | TGAGTATTTGCTCTGCGTCCT | CCCAGGTGGTCTTAAATGTCCT | 3984 | 55 |
| Exon 9 | GCCTTCCGCTTCTATGGTCC | TGAGTGAGCAGGATTTTGGCA | 1680 | 60 |
| Sequencing primers | | |  |  |
| Region | Sequencing primer (5’–3’) |  |  |  |
| Promoter | CCTTAGAAAGAGAACAAGAGACTTC |  |  |  |
| Exon 1 | CACTTTCAAATACAGGACCTCG |  |  |  |
| Exon 3 and Exon 4 | ATTTCTCTGGGCAGTTTTCAAGTG |  |  |  |
| Exon 5 | ATGCCTTCAAACACTCAACC |  |  |  |
| Exon 6 and Exon 7 | TGAGTATTTGCTCTGCGTCCT |  |  |  |
| Exon 9 | GCCTTCCGCTTCTATGGTCC |  |  |  |


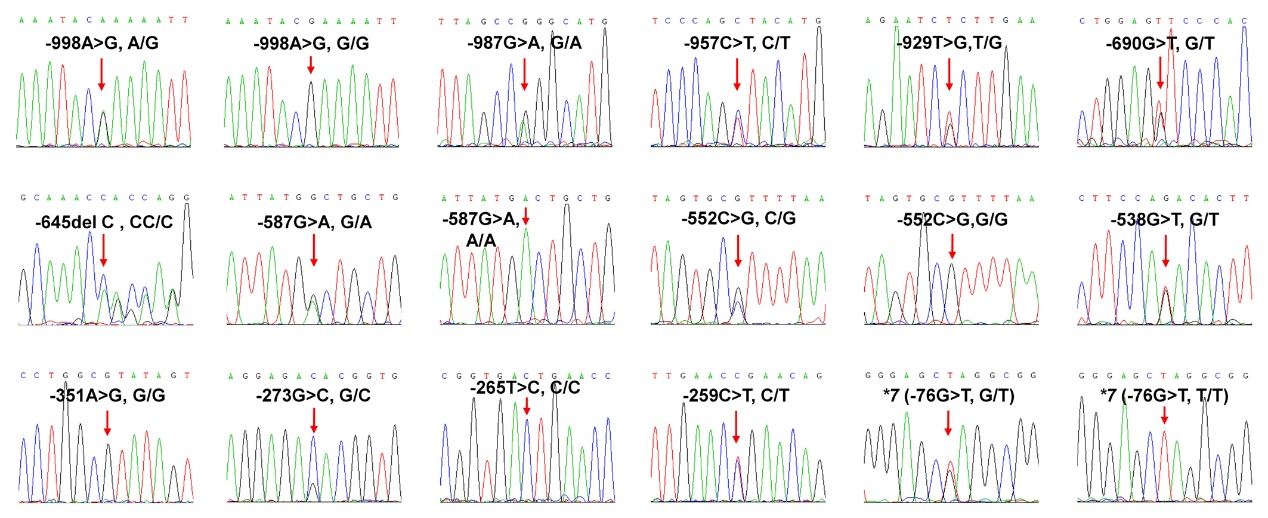


**Figure S1 Sequencing results of *CYP2J2* in subjects carrying promoter variants.**

The results of *CYP2J2*7* and 13 *CYP2J2* promoter variants are shown. *CYP2J2* promoter variants and their genotypes are labeled with red arrows.
